# IκB_NS_-deficiency protects mice from fatal *Listeria monocytogenes* infection by blunting pro-inflammatory signature in Ly6C^high^ monocytes and preventing exaggerated innate immune responses

**DOI:** 10.3389/fimmu.2022.1028789

**Published:** 2022-12-22

**Authors:** Sarah Frentzel, Andreas Jeron, Alexander Pausder, Olivia Kershaw, Julia Volckmar, Ingo Schmitz, Dunja Bruder

**Affiliations:** ^1^ Immune Regulation Group, Helmholtz Centre for Infection Research, Braunschweig, Germany; ^2^ Institute of Medical Microbiology and Hospital Hygiene, Infection Immunology Group, Health Campus Immunology, Infectiology and Inflammation, Otto-von-Guericke University Magdeburg, Magdeburg, Germany; ^3^ Department of Veterinary Medicine, Institute of Veterinary Pathology, Free University Berlin, Berlin, Germany; ^4^ Dept. of Molecular Immunology, Ruhr University Bochum, Bochum, Germany

**Keywords:** IκB_NS_, *Listeria monocytogenes*, *in vivo* infection, hyper-inflammation, myeloid cells, innate immunity

## Abstract

IκB proteins regulate the inhibition and activation of NF-κB transcription factor complexes. While classical IκB proteins keep NF-κB complexes inactive in the cytoplasm, atypical IκB proteins act on activated NF-κB complexes located in the nucleus. Most of the knowledge regarding the function of IκB proteins has been collected *in vitro*, while far less is known regarding their impact on activation and regulation of immune responses during *in vivo* infections. Combining *in vivo Listeria monocytogenes* (Lm) infection with comparative *ex vivo* transcriptional profiling of the hepatic response to the pathogen we observed that in contrast to wild type mice that mounted a robust inflammatory response, IκB_NS_-deficiency was generally associated with a transcriptional repression of innate immune responses. Whole tissue transcriptomics revealed a pronounced IκB_NS_-dependent reduction of myeloid cell-associated transcripts in the liver together with an exceptionally high *Nfkbid* promoter activity uncovered in Ly6C^high^ inflammatory monocytes prompted us to further characterize the specific contribution of IκB_NS_ in the inflammatory response of monocytes to the infectious agent. Indeed, Ly6C^high^ monocytes primed during Lm infection in the absence of IκB_NS_ displayed a blunted response compared to wild type-derived Ly6C^high^ monocytes as evidenced by the reduced early expression of hallmark transcripts of monocyte-driven inflammation such as *Il6*, *Nos2* and *Il1β*. Strikingly, altered monocyte activation in IκB_NS_-deficient mice was associated with an exceptional resistance against Lm infection and protection was associated with a strong reduction in immunopathology in Lm target organs. Of note, mice lacking IκB_NS_ exclusively in myeloid cells failed to resist Lm infection, indicating that the observed effect was not monocyte intrinsic but monocyte extrinsic. While serum cytokine-profiling did not discover obvious differences between wild type and IκB_NS_^-/-^ mice for most of the analyzed mediators, IL-10 was virtually undetectable in IκB_NS_-deficient mice, both in the steady state and following Lm infection. Together, we show here a crucial role for IκB_NS_ during Lm infection with IκB_NS_-deficient mice showing an overall blunted pro-inflammatory immune response attributed to a reduced pro-inflammatory signature in Ly6C^high^ monocytes. Reduced immunopathology and complete protection of mice against an otherwise fatal Lm infection identified IκB_NS_ as molecular driver of inflammation in listeriosis.

## Introduction

Infections trigger inflammatory responses in the infected host that are orchestrated by a complex interplay of innate and adaptive immune cell subsets. Activation, differentiation and effector functions of immune cells are mediated by transcription factors such as the ubiquitously expressed NF-κB transcription factors, that regulate expression of a plethora of genes involved in inflammation processes (1). A protein family of classical and atypical inhibitory IκB proteins regulates the inhibition and activation of NF-κB transcription factors. In resting cells, classical IκBs are bound to NF-κB complexes, thereby retaining them in the cytoplasm by masking their nuclear localization domain and consequently keeping them in an inactive form. Upon stimulation, the activation of a multi-kinase complex leads to the phosphorylation and rapid proteasomal degradation of IκB proteins. The released NF-κB complex translocates into the nucleus and binds to the κB binding motifs in the DNA (2). Within the nucleus, the transcriptional NF-κB activity is further modulated by atypical IκBs which have effects on the regulation of NF-κB dimer exchange, recruitment of histone modifying enzymes or the stabilization of NF-κB dimer binding to DNA (3).

The atypical inhibitory IκB protein IκB_NS_ was first identified in the context of negative selection of thymocytes (4). Yet, IκB_NS_-deficient mice exhibit normal numbers and distribution of conventional T cell subpopulations in thymus, spleen and lymph nodes. However, IκB_NS_-deficient CD8^+^ T cells show reduced proliferation and secretion of interleukin-2 (IL-2) and interferon-gamma (IFN-γ) upon *in vitro* T cell receptor stimulation (5). Furthermore, IκB_NS_ is important for the development of thymus-derived naturally occurring and induced Foxp3^+^ regulatory T cells (Treg). Interaction of IκB_NS_ with the p50/c-Rel NF-κB dimer bound to the conserved non-coding sequence -3 (CNS3) is needed for full transcriptional activation of the *Foxp3* locus (6). However, despite reduction of mature Treg cell numbers in IκB_NS_-deficient mice, IκB_NS_ seems dispensable for their functionality in steady state (6). In addition, IκB_NS_ is crucial for the differentiation of CD4^+^ T cells into Th17 effector cells. IκB_NS_ is highly expressed in Th17 cells and in its absence, reduced frequencies of IL17-producing T cells are observed *in vitro* under Th17 polarizing conditions and in *Citrobacter rodentium* infection *in vivo*. In the latter, IκB_NS_-deficient mice exhibit increased bacterial burden, but reduced tissue pathology due to impaired Th17 effector cell induction (7, 8). Moreover, IκB_NS_-deficiency leads to a significantly reduced activation of CD4^+^ effector T cells with impaired antigen-specific proliferation and secretion of effector cytokines in response to *in vivo* infection with *Listeria monocytogenes* (9). Beside the impact of IκB_NS_ on T cells, several studies revealed that IκB_NS_ influences the development and function of B lymphocytes (10–12).

In contrast to adaptive immune cells, only limited knowledge exists regarding its impact on innate immune cell activation and phenotype. Here, IκB_NS_ was shown to be constitutively expressed in colonic *lamina propia* macrophages where it is thought to have suppressive functions on inflammatory responses in the intestine. More precisely, it suppresses lipopolysaccharide (LPS)-induced IL-6 production in macrophages through a constitutive binding to the *Il6* promoter together with p50 (13). Additionally, IκB_NS_ selectively inhibits the expression of IL-6, IL-12p40 and IL-18 in macrophages upon LPS stimulation and IκB_NS_-deficient mice succumbed to LPS-induced endotoxin shock, possibly due to sustained production of the pro-inflammatory mediators IL-6 and IL-12p40 (14). So far, most studies related to the immunological function of IκB_NS_ confined to certain cell types and specific *in vitro* conditions and are lacking thorough parallel measurement of IκB_NS_ expression in various immune cell subsets together with its function during natural *in vivo* orchestrated immune responses.

The intracellular pathogen *Listeria monocytogenes* (Lm) is widely used as a model organism to study various aspects of innate and adaptive immunity in mice. Innate immunity to Lm involves the secretion of IFN-γ by natural killer cells that in turn contributes to macrophage activation. Tumor-necrosis factor (TNF)-α is also necessary for the primary defense against Lm infection and mice lacking both, IFN-γ and TNF-α, rapidly succumb to Lm infection (15). Macrophages and neutrophils are both key players in eliminating Lm within the infected host, and recruited monocytes are crucial for mounting pro-inflammatory immune responses which mediates bacterial clearing through production of nitric oxide (15). While the innate immune response is important for recognition and early control of bacteria, CD4^+^ and CD8^+^ T cells confer clearance of bacteria and mediate long-term protection against re-infection with the pathogen (16). IκB_NS_ exhibits dual role as a suppressor and promoter of transcription of NF-κB target genes. This together with the facts, that the immune response to Lm orchestrates a hallmark of NF-κB targets in many innate immune cell subsets (17) and Lm pathogenicity and *in vivo* distribution has been shown previously to depend on NF-κB signaling (18) prompted us to apply Lm infection in mice to study in more detail the role of IκB_NS_ in early antibacterial immunity during *in vivo* infection.

## Material and methods

### Animals

IκB_NS_^+/+^ and IκB_NS_-/- mice (B6.129/SV-Nfkbid^(tm1Clay)^) (5, 6), were bred and maintained under specific pathogen-free conditions at the animal facilities of the Helmholtz Centre for Infection Research (HZI) Braunschweig, Germany and the University Hospital Magdeburg, respectively. Nfkbid^lacZ^ (Nfkbid^tm1a(EUCOMM)Wtsi^) reporter mice were obtained from the EUCOMM consortium. Nfkbid^LacZ^ mice have a promoter-less cassette containing a LacZ coding sequence, which encodes ß-galactosidase and is inserted into one allele of the *Nfkbid* locus allowing the indirect detection of *Nfkbid* promoter activity through assessment of enzymatic ß-galactosidase activity. This can be achieved by using surrogate galactose substrate analogue with fluorescent features like Fluorescein Di-D-Galactopyranoside (FDG), which releases fluorescein dye upon lacZ cleavage. Though Nfkbid^LacZ^ mice only have one functional allele for the *Nfkbid* gene, they do not exhibit any specific phenotype and show a normal development under steady state conditions.

Nfkbid^ΔLysM^ mice were generated by crossing homozygous Nfkbid^tm1c(EUCOMM)Wtsi^ (Nfkbid^flox/flox^) mice (9) with heterozygous B6.129P2-*Lyz2^tm1(cre)Ifo^*/J (LysM-Cre) mice (19). An in-depth characterization of these mice will be described elsewhere.

For infection experiments female mice at the age of 10-20 weeks were used. We performed all analysis in mice of the same sex since it was shown previously that there are sex-related differences in susceptibility of mice to Lm (20) that might potentially overlay IκB_NS_-dependent differences in immunity to Lm infection. All experiments were performed in accordance with the institutional guidelines and were approved by the Landesamt für Verbraucherschutz, Sachsen-Anhalt and the Landesamt für Verbraucherschutz und Lebensmittelsicherheit, Niedersachsen.

### Bacterial infection

*Listeria monocytogenes* (strain 10403S) were grown overnight (37°C, 180 rpm) in BHI broth (BD Biosciences). A 1:5 dilution was prepared with fresh BHI and after 3 h bacteria were harvested and diluted in sterile PBS to establish an infection dose of 1x10^5^ CFU/mouse and 100 µL was injected into the tail vein of mice. The infection dose was controlled by plating serial dilutions of the inoculum on BHI agar plates and counting the colonies after incubation at 37°C for 24 h. To determine CFU in spleen and livers, organs were homogenized in 0.2% IGEPAL CA-630 (Sigma-Aldrich) lysis buffer and serial dilutions were plated on BHI agar plates to quantify colonies after incubation at 37°C for 24 h.

### Histopathology

Uninfected and Lm infected WT and IκB_NS_^-/-^ mice were euthanatized by inhalation of CO_2_ on day 0 and 4 dpi. Spleens and livers were carefully removed and shortly washed in PBS. Afterwards, the tissues were fixed in 4% paraformaldehyde (Sigma-Aldrich), paraffin embedded, 5 µm sections were cut and stained with hematoxylin and eosin. Histopathological changes were graded in a blinded manner regarding the quality and quantity of inflammation and necrosis as described elsewhere (21).

### Determination of ALT

Mice were sacrificed by CO_2_-euthanasia and blood was obtained by puncture of the heart with a 25 gauge needle on a 1 mL syringe. Blood samples were incubated first for 20 min at RT followed by incubation for 20 min at 4°C. Afterwards, samples were spun down (14000 rpm) at 4°C for 5 min and serum-containing supernatant was collected and stored at -20°C until further analysis. For analysis of the serum alanine transaminase (ALT) level, 32 μL serum was pipetted on a Refloton^®^ test stripe and measured on the spectrophotometer Reflovet Plus (Roche).

### Cell preparation

Spleens were squeezed through 100 µm cell strainers and washed with PBS (300xg, 10 min, 4°C) followed by erythrocytes lysis using hypo-osmolaric NaCl buffers (0.2% NaCl, 1.6% NaCl). Lysis was stopped by filling up with PBS and centrifugation by 300xg, 10 min and 4°C. The supernatant was discarded and the pellet was resuspended in sterile PBS and splenocytes were passed through a 30°µm cell strainer and stored on ice until further analysis: Liver cells were separated using the gentleMACS device (Miltenyi Biotec) and the Liver Dissociation Kit (Miltenyi Biotec) according to manufacturer recommendations. Afterwards erythrocytes lysis was performed by hypo-osmolaric NaCl buffers as described for splenocytes. Cells were passed through a 30 µm cell strainer and a density gradient centrifugation was performed with Percoll (GE Healthcare, ϱ=1.041 g/mL, 360xg, 20 min, RT). Cells were washed twice with PBS and resuspended in PBS and stored on ice until further analysis.

### Flow cytometric analyses

Cells were incubated with anti-CD16/32 (Fc-block, BioLegend) to prevent unspecific binding of antibodies and stained with Fixable Viability Dye (eFlour506, eBioscience) to exclude dead cells. After washing, cells were incubated with an antibody mixture containing Ly6C-PerCP-cy5.5 (HK1.4; Biolegend), CD24-PE (M1/69, Biolegend), CD8-PE-Cy5 (53-6.7, Biolegend), F4/80-PE-Cy7 (BM8, Biolegend), CD11c-APC (N418, Biolegend), Ly6G-AlexaFluor700 (1A8, Biolegend), CD11b-APC-Cy7 (M1/70, Biolegend), CD64-BV421 (X54-5/7.1, Biolegend), CD45-BV605 (30-F11, Biolegend) and IA/IE-BV650 (M5/114.15.2, Biolegend) for 10 min at 4°C in the dark. Gating of different immune subsets were performed as described elsewhere (22) Afterwards cells were washed with PBS and resuspended in an appropriate volume of FACS buffer.

### Measurement of LacZ activity

To quantify the ß-galactosidase activity of the lacZ reporter within cells of the Nfkbid^lacZ^ mice the FluoReporter™ lacZ Flow Cytometry kit (Thermo Fisher) was used according to manufacturer recommendations. The prepared single cell suspension from the respective organs was adjusted to 10^7^ cells/mL and 55 μL of the cell suspension was transferred into a 5 mL flow cytometer tube. The FDG reagent provided by the kit was thawed on ice and a 2 mM working solution was prepared by diluting the thawed FGD reagent 10-fold with deionized water. The working solution was briefly warmed at 37°C and vortexed to obtain a homogenous solution. Afterwards the prepared cell suspension was loaded with FDG by adding 55 μL of the prewarmed 2 mM working solution to the cell suspension. Through the hypotonic shock the FDG can enter the cells and can be potentially hydrolyzed by the cytoplasmic reporter ß-galactosidase enzyme (lacZ). After incubation for exactly 1 min at 37°C, the FDG loading was stopped by adding 1 mL ice-cold PBS (Gibco) containing 4% (v/v) FCS (PAN Biotech) and 10 mM HEPES (Gibco). The suspension was centrifuged (5 min, 300xg, 4°C) and cell surface staining was performed as described before. For all staining steps, the cells were kept on ice and protected from light. Flow cytometric acquisition was performed on the same day using either LSR-Fortessa (BD Biosciences) or Attune NxT Flow Cytometer (Thermo Fisher Scientific).

### Flow cytometric cell sorting

Spleens and livers from either WT, IκB_NS_^-/-^ or Nfkbid^ΔLysM^ mice were sampled on day 3 post Lm infection. Splenocytes and cells from liver tissue were collected as described previously. Splenocytes were pre-sorted by using magnetic cell separation (autoMaCS, Miltenyi Biotec) to eliminate B cells and T cells according to manufacturer recommendations. Cells from 5 individual uninfected as well as Lm infected WT and IκB_NS_^-/-^ mice were pooled per genotype to ensure sufficient cell numbers after FACS-based cell sorting. For sorting of the inflammatory monocytes the cells were stained as described above excluding antibody against CD8, but instead in addition with CD115-APC (AFS98, BioLegend). Sorting was performed on a BD FACS Aria III (BD Biosciences) and sorted cell fractions were resuspended in 350 µL RLT buffer (Qiagen) and stored at -20°C until further use.

### RNA preparation and real-time RT-PCR

RNA was isolated using the RNeasy Mini Kit (Qiagen) according to manufacturer instructions. DNA was removed by using the RNase-Free DNase set (Qiagen) and RNA was finally eluted in 50 µL nuclease-free ultrapure water. The RNA concentration was measured by the NanoDrop ND-1000 spectrophotometer (Thermo Fisher Scientific) and equal amounts (around 1 µg) were used for cDNA synthesis in a reverse transcription reaction. cDNA was used as a template for real-time PCR using SYBR Green I (Roche). Quantitative RT-PCRs were run in duplicates using the LightCycler 480 system II (Roche) using the following primers: *Il6_*fwd: TCTAATTCATATCTTCAACCAAGAGG, *Il6_rev*: TGGT CCTTAGCCACTCCTTC; *Nos2_fwd*: TGGAGGTTCTGG ATGAGAGC, *Nos2_rev*: AATGTCCAGGAAGTAG GTGAGG; *Il1β_fwd*: TGAAATGAAAGACGGCA CACC, *Il1β_rev*: TCTTCTTTGGGTATTGCTT GG; *Rps9_fwd*: CTGGACGAGGGCAAGATGAAGC, *Rps9_rev*: TGA CGTTGGCGGATGAGCACA; *Actin-β_fwd*: CTTCTTT GCAGCTCC, *Actin-β_rev*: TCCTTCTGACCCATTCCCAC. *Rps9* and *Actin-β* were used as housekeeping genes for normalization and the relative expression of the target genes were calculated by the LightCycler 480 software according to the ΔΔCt method.

### Microarray analysis

RNA from whole liver homogenates was isolated and purified using the RNeasy Mini Kit (Qiagen) and the RNase free DNase kit (Qiagen) according to manufacturer recommendations. Per analyzed point in time following infection the RNA concentration of samples from 3-5 WT and IκB_NS_^-/-^ mice was measured with a photospectrometer (NanoDrop) and equal amounts of RNA from each sample were pooled to obtain a representative sample mix integrating biological variances. Further sample preparation, amplification, fragmentation, microarray hybridization, staining and scanning were performed by the Genome Analytics Group at Helmholtz Centre for Infection Research (Braunschweig, Germany). Liver samples were analyzed with the GeneChip^®^ Mouse Gene 2.0 ST microarray (Affymetrix) according to manufacturer instructions. In total 8 microarrays reflecting the following conditions were performed: WT: d0, d2, d3, d4; IκB_NS_^-/-^: d0, d2, d3, d4. Sorted liver monocytes were analyzed by Clariom S Mouse microarray (Affymetrix).

Microarray raw data were processed by Transcriptome Analysis Console (TAC, version 4.0, Thermo Fisher Scientific). Data were summarized, log_2_-transformed and quantile-normalized with RMA algorithm including background subtraction or analyzed with the SST-RMA algorithm (monocyte data). A percentile filter was applied to the microarray data in a way, that only transcripts with signal intensity above the 20^th^ percentile of all signal intensities in a given microarray were retained and only if this is the case in at least one microarray. Differentially regulated genes in liver samples were identified as follows. Per genotype the comparisons: d2 vs. d0, d3 vs. d0, d4 vs. d0 were considered, resulting in together 6 different conditions. Next, a list of genes was compiled only containing entries with a fold change of differential gene expression of > ± 3 in at least one out of the 6 previous conditions. Scatter dot plots were generated using GraphPad Prism Software v5 (La Jolla, CA, USA). K-means cluster analysis of z-score transformed microarray data of differentially regulated genes was carried out by Genesis Software (version 1.8.1, 23). Gene Ontology enrichment analysis was performed using Cytoscape software and ClueGO plugin (24).

### Cytometric bead assay

Mice were sacrificed and blood was obtained by puncture of the heart. Blood samples were incubated for 30 min at RT followed by 30 min incubation at 4°C. After centrifugation (14000 rpm, 5 min) supernatant was collected and stored at -20°C until further analysis. Serum cytokines were quantified in 1:2 diluted samples using a cytometric bead array (LEGENDplex™ Mouse Inflammation cytokine panel (13-plex), BioLegend) according to the manufacturer’s recommendations. Data acquisition was performed on Attune NxT Flow Cytometer (Thermo Fisher Scientific) and data analysis by using the software provided by the Legendplex (Biolegend).

### Statistics

Results are expressed as mean ± standard error of the mean (SEM). Statistical analyses were performed with the GraphPad Prism 5.4 Software (La Jolla, CA, USA) and a p-value below 0.05 was considered significant. 

## Results

### IκB_NS_-deficiency broadly affects the hepatic gene expression profile during *Listeria monocytogenes* infection

**Figure 1:**
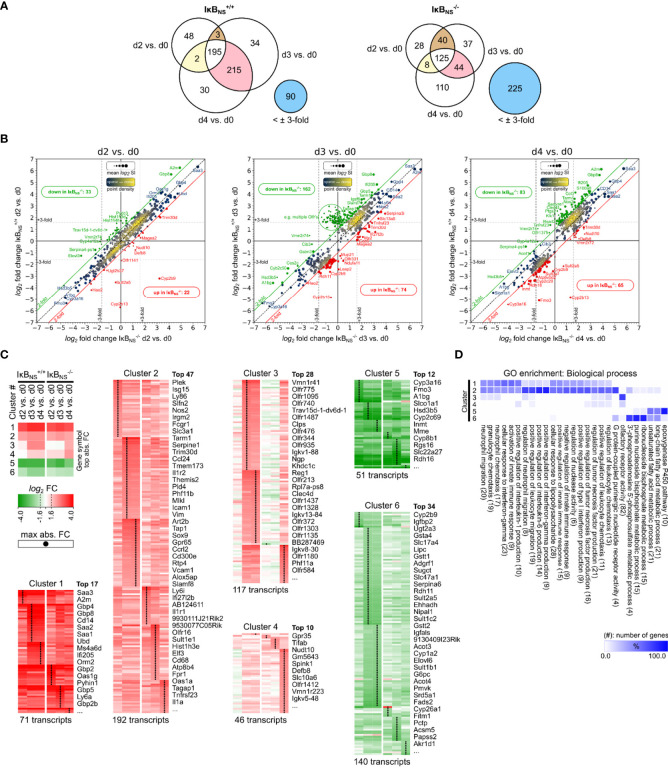
Transcriptome analysis of Listeria infected IκB_NS_^-/-^ and wild type livers. WT and IκB_NS_^-/-^ mice were infected with 10^5^ CFU LM i.v. or left untreated (d0). Mice were sacrificed on day 2, 3 and 4 post infection, liver homogenates were prepared and sampled for total RNA isolation. Equal amounts of RNA from n = 3-5 mice per genotype and sampling time were pooled per experimental group and applied to Affymetrix Gene 2.0 ST microarray analysis. **(A)** Venn diagrams of 617 annotated transcripts differentially regulated at least ±3-fold in reference to d0 in IκB_NS_^-/-^ or WT livers on d2, d3 or d4. Stated integers refer to the number of transcripts with fold change of at least ±3-fold in the indicated comparison and the extend of gene symbol overlap between them. Blue circles refer to transcripts not regulated IκB_NS_^-/-^ or WT liver d2, d3, d4 comparisons, respectively. **(B)** log_2_/log_2_ fold change scatter plots of conditions shown in a), comparing log_2_ fold changes of the 617 transcripts between IκB_NS_^-/-^ (x-axis) and WT livers (y-axis) per analysis day post infection. Red and green diagonal threshold lines refer to log_2_ fold change differences of at least 2-fold. Framed red/green integers refer to the number of transcripts above/below this threshold in IκB_NS_^-/-^ livers. Dashed vertical/horizontal threshold lines refer to the fold change critirion ( ± 3-fold) applied in the comparisons shown in a). Gene symbols of selected transcripts are stated (red: up in IκB_NS_^-/-^, green: down in IκB_NS_^-/-^). **(C)** log_2_ fold changes of conditions in a) for the 617 transcripts were color coded and k-means clustered (k = 6). Shown gene symbols in each cluster are ranked by average absolute fold change and only the top n symbols are stated. Total numbers of transcripts per cluster are stated below. Black points in clusters indicate condition with maximal absolute fold change per transcript. Top left: Legend with cluster summary, representing average log_2_ fold changes per condition and cluster. **(D)** Gene ontology (GO) enrichment analysis for GO category “Biological process” showing terms with FDR < 0.05 and GO level ≥ 8. Percent of genes falling into clusters shown in c) in reference to the total number of mapped genes within overrepresented GO terms were calculated, hierarchically sorted and color coded. Total number of mapped genes per GO term is stated in brackets.

To study dependency of innate immune activation on IκB_NS_ in a physiological *in vivo* setting, we performed Lm infections in IκB_NS_-sufficient (IκB_NS_^+/+^, wild type) *versus* IκB_NS_-deficient (IκB_NS_^-/-^) mice. The liver constitutes one of the major target sites for Lm replication. As such, inflammatory immune activation and subsequent immune cell recruitment and liver pathology are induced early on after infection. To get a focused starting point regarding the overall contribution of IκB_NS_ in the transcriptional regulation of acute inflammatory responses *in vivo*, we performed unbiased transcriptional profiling of whole livers from IκB_NS_^+/+^ and IκB_NS_^-/-^ mice directly prior (day 0) and at different times (day 2, day 3 and day 4) post infection. To compare the kinetics of the transcriptional response in WT and IκB_NS_^-/-^ mice, microarray data were analyzed for differential gene expression at different times post infection in reference to the uninfected control. A filtered list was compiled including differentially expressed genes with a fold change of at least ±3 in at least one out of the following 6 comparisons: 1.) IκB_NS_^+/+^ d2 vs. d0; 2.) IκB_NS_^+/+^ d3 vs. d0; 3.) IκB_NS_^+/+^ d4 vs. d0; 4.) IκB_NS_^-/-^ d2 vs. d0; 5.) IκB_NS_^-/-^ d3 vs. d0; 6.) IκB_NS_^-/-^ d4 vs. d0. This list contained in total 617 (**Supplementary table 1**) unambiguously annotated transcripts of which 527 were found to be regulated in IκB_NS_^+/+^ (**Figure 1A**, left) and 392 transcripts found to be regulated in IκB_NS_^-/-^ mice (**Figure 1A**, right) on day 2, 3 or 4 post infection.

The Venn diagrams in **Figure 1A** summarize numeric differences in differentially regulated genes between the genotypes. In general, transcriptional alterations in livers of wild type mice were most pronounced on day 3 and day 4 post Lm infection. Interestingly, the overall number of regulated transcripts in IκB_NS_^-/-^ livers was clearly lower, indicating a blunted early transcriptional response to Lm infection in IκB_NS_^-/-^ mice (**Figure 1A**). Likewise, comparison of the two genotypes regarding the number of transcripts being regulated both on day 3 and day 4 post Lm infection indicates an overall reduced and delayed transcriptional response to Lm infection in the absence of IκB_NS_. Of note, 225 transcripts which are regulated in at least one of the wild type conditions, are not regulated to a similar extend in IκB_NS_^-/-^ livers. On the other hand, in wild type livers only 90 transcripts are not regulated and hence are exclusively regulated in IκB_NS_ knockout mice.

**Figure 1B** indicates log_2_ fold changes from the conditions stated in the Venn diagrams, but for each day post infection comparing differential expression in IκB_NS_^-/-^ *versus* wild type livers in reference to d0. Here, the consequences of IκB_NS_-deletion, superimposed with the course of the Lm induced inflammatory response, becomes evident. On day 2 post infection, IκB_NS_-deficiency only marginally affects the transcriptional profile in the Lm infected liver. However, numbers of deviating transcripts dramatically increase by day 3 post infection and remain elevated on day 4. Of note, comparison of both genotypes regarding their default conditions on day 0 revealed no relevant differential gene expression (data not shown).

IκB_NS_-dependency of the early transcriptional response to Lm infection was further analyzed by performing k-means clustering (k = 6) of log2 fold changes, followed by time-sequential sorting of resulting gene clusters according to their averaged expression profiles (**Figure 1C**). Next, gene clusters were analyzed by Gene Ontology (GO) analysis for statistically over-represented terms in the “Biological process” GO category. Cluster-specific GO enrichment results were hierarchically clustered to reveal groups of functional attributions of transcripts within each of the 6 gene clusters (**Figure 1D**).

Both, clusters 1 and 2 group genes that are strongly induced upon infection compared to the uninfected condition (d0), with cluster 1 containing transcripts with the highest positive fold change. Of note, transcripts in both clusters followed a similar pattern with continuous upregulation in both genotypes from day 2 to 4 post infection. Thus, it is reasonable to regard the observed pattern as a typical response to Lm infection that is mostly independent of the IκB_NS_ genotype. This involves terms related to cellular response to LPS, leukocyte (e.g. neutrophils) migration and chemotaxis, regulation of innate immune responses and more specifically, regulation of type I IFN, TNF-α, IL-6 and IL-1 production, and response to IFN-γ signaling.

Cluster 3 is the most interesting cluster with regard to genotype-dependent differences in Lm infected livers. Transcripts in this cluster are strongly upregulated on day 3 and 4 in IκB_NS_^+/+^ livers. This response is severely delayed in IκB_NS_^-/-^ livers where it only becomes evident on day 4 post infection. Moreover, the level of gene induction is generally lower in knockout livers compared to wild type mice for most of the identified transcripts. Functional attributions show strong enrichment of olfactory receptor activity and minor enrichments in GO terms like regulation of TNF-α production, leukocyte and granulocyte chemotaxis/migration. Cluster 4 also shows genotype-related differences in expression profiles, which mainly refer to d0 in wild type and day 4 in knockout livers. IκB_NS_^-/-^ livers expression of cluster 4 transcripts is delayed with robust induction only by day 4 post infection. Functional attributions reveal minor enrichments in the regulation of TNF-α production, leukocyte chemotaxis/migration, regulation of IL-1 production and olfactory receptor activity. Cluster 5 and cluster 6 contain transcripts downregulated following infection. Functionally enriched terms in both clusters mainly relate to liver metabolic processes.

Having a closer look to those transcripts whose expression showed a particularly strong dependency on IκB_NS_, we made the striking observation that many of them are associated with myeloid cell phenotype and function. More specifically, *Gbp8* (Guanylate-binding protein 8) was ~ 50-fold induced in the liver of IκB_NS_^+/+^ mice day 3 post infection compared to only ~13-fold induction in IκB_NS_^-/-^ livers. In the same line, *Ifi205* (Interferon-activable protein 205-A) was induced 28-fold in IκB_NS_^+/+^ mice compared to 9-fold in IκB_NS_^-/-^ mice. Further examples for myeloid cell-associated genes whose expression heavily depended on IκB_NS_ are *Ccl3* (Chemokine C-C motif ligand 3, d4 WT:17-fold, d4 KO: 3-fold), *Cd14* (d4 WT: 20-fold, d4 KO: 12-fold), *Retnlg* (Resistin like gamma, d4 WT: 15-fold, d4 KO: 6-fold), *Tarm1* (T cell-interacting activating receptor on myeloid cells 1, d3 WT: 12-fold, d3 KO: 4-fold), *Csf2rb2* (Colony stimulating factor 2 receptor beta 2, d4 WT: 6-fold, d4 KO: 2-fold), *Clec4d* (C-type lectin domain family 4 member d, d4 WT: 6-fold, d4 KO: 1.4-fold) and *Nos2* (Inducible nitric oxide synthase 2, d4 WT: 8-fold, d4 KO: 3-fold).

Taken together, unbiased microarray analysis gave compelling insights into the dependency of the early Lm-induced transcriptional response of the liver on IκB_NS_. Lm-mediated induction of inflammatory responses encompasses leukocyte attraction/migration, interferon production/sensing, TNF-α, IL-6 and IL-1 production as well as reduction of liver metabolic processes. Interestingly, lack in IκB_NS_ differentially affects certain aspects of the inflammatory response, but is generally associated with a transcriptional repression or delay compared to wild type mice. Moreover, we identified a striking reduction of myeloid cell-associated transcripts in mice lacking IκB_NS_. Since Lm-infection is well known to trigger an immune response in myeloid cells of the liver (25), the severely impaired induction of numerous myeloid cell-associated genes in mice lacking IκB_NS_ prompted us to hypothesize, that in particular myeloid cells may depend on IκB_NS_ to fulfill their inflammatory function in the early innate immune response to infection.

### Hepatic Ly6C^high^ monocytes exhibit high basal *Nfkbid* expression

**Figure 2:**
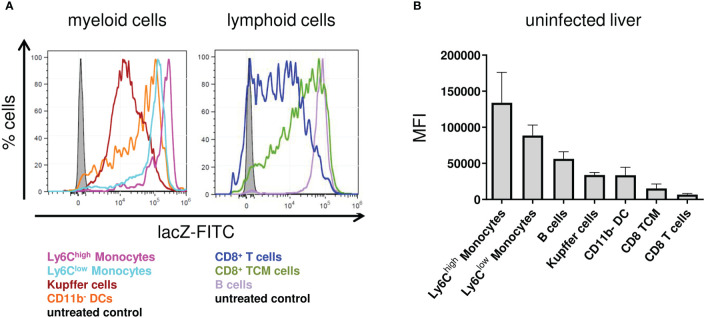
Nfkbid promoter activity in different immune cell subsets in the liver of Nfkbid^lacZ^ reporter mice. Nfkbid^lacZ^ reporter mice (n = 4) were sacrificed, livers were sampled and total leukocytes were isolated. FACS analyses for the differentiation of stated immune cells subsets was performed together with intracellular lacZ FDG reporter assay. **(A)** Representative histogram overlays of fluorescein reporter fluorescence intensities in myeloid (left) and lymphoid (right) cellular subsets. **(B)** Numeric representation of fluorescein reporter fluorescence intensities. Data represent mean ± SD of median fluorescence intensity.

Since IκB_NS_-expression level was shown to be inducible upon stimulation in macrophages (13, 14), we wanted to address if there is a basal expression level of IκB_NS_ in different immune cell subsets from the liver. Therefore, we profiled the IκB_NS_ expression pattern in various myeloid and, as comparison, in lymphoid cell subsets. To this end, we made use of a *Nfkbid* reporter mouse (Nfkbid^LacZ^) we recently described, which allows the evaluation of *Nfkbid* promoter activity as a surrogate for IκB_NS_ protein expression by means of a flow cytometry-based reporter assay correlating *Nfkbid* promoter activity to lacZ enzyme expression. Strikingly, we indeed identified a particularly high basal IκB_NS_ expression in myeloid cell subsets in the liver and here especially in Ly6C^high^ inflammatory monocytes that exceeded the expression level observed in Ly6C^low^ monocytes (**Figure 2A**). A similar *Nfkbid* promoter activity pattern was found in splenic leukocytes from naive mice (data not shown), suggesting *Nfkbid* promoter activity in immune cells to be independent from the organ-specific microenvironment.

**Figure 3:**
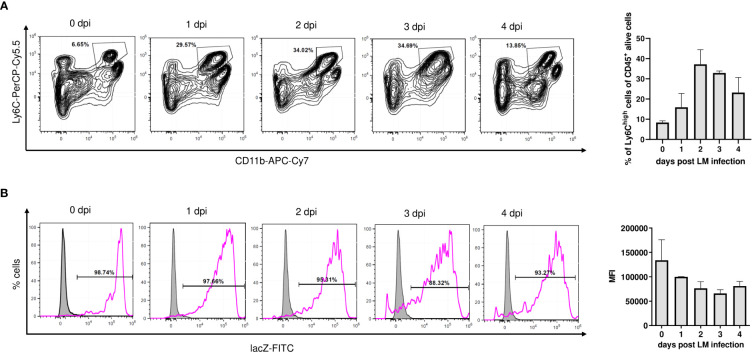
Nfkbid promoter activity in Ly6C^high^ monocytes of Nfkbidlacz reporter mice during Lm infection. Nfkbid lacZ reporter mice (n = 4-5 mice per time point) were infected with LM (3 x 10^4^ CFU/mouse) and sacrificed on indicated time points. Livers were sampled, total leukocytes were isolated and analyzed by FACS in combination with lacZ FDG reporter assay. CD11b^+^Ly6^high^ inflammatory monocytes in the liver were pre-gated on viable singlet CD45^+^ cells and Nfkbid promoter activity was determined as median fluorescence intensity of fluorescein reporter dye. **(A)** Representative contour plots, showing gating and frequency of CD11b^+^Ly6^high^ inflammatory monocytes (left) and summarized frequency data representing mean ± SD (right). **(B)** Representative histograms of lacZ reporter fluorescence intensity in gated CD11b^+^Ly6C^high^ inflammatory monocytes (pink) and reporter negative control (grey). Summarized Nfkbid lacZ reporter intensity representing mean ± SD is shown on the right. Shown data are from one out of two independent experiments with similar results.

Since activation-dependent changes in IκB_NS_ expression have been reported for lymphocytes (4, 8), we next addressed how Lm-induced immune activation would affect IκB_NS_ expression levels in monocytes. Well in line with the liver transcriptome showing induction of hallmark mediators involved in monocyte recruitment to the site of infection, we observed a rapid increase of Ly6C^high^CD11b^+^ inflammatory monocytes in the liver of Nfkbid^LacZ^ reporter mice upon Lm infection. After a peak on day 2 post infection, the frequency of Ly6C^high^CD11b^+^ cells was gradually decreasing until day 4 post infection (**Figure 3A**). Despite dynamic fluctuation of the Ly6C^high^ monocyte frequency that is driven by migration as well as apoptotic processes and continuous phenotypical advancement of monocytes within the liver itself, we found *Nfkbid* expression to remain constantly high during infection (**Figure 3B**). This may suggest that IκB_NS_ is highly relevant for the development, activation, and/or function of Ly6C^high^ monocytes. This assumption is supported by data from whole liver microarray analysis showing a blunted pro-inflammatory response especially of genes involved in monocyte function in the absence of IκB_NS_ (**Figure 1**).

### IκB_NS_-deficiency is associated with a blunted pro-inflammatory response in Ly6C^high^ monocytes during Lm infection

**Figure 4:**
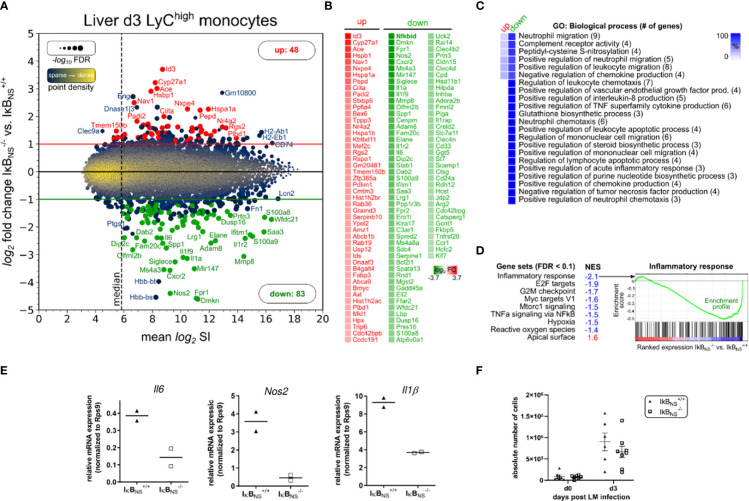
Transcriptome analysis of Ly6C^high^ monocytes sorted from IκB_NS_^-/-^ and wild type livers 3 days post Lm infection. WT and IκB_NS_^-/-^ mice (n = 3-5 per group, from two independent experiments) were infected with 3×10^4^ CFU LM i.v., sacrificed on day 3 post infection, liver tissue was enzymatically digested, total leukocytes were isolated using Percoll density gradient centrifugation, Ly6C^high^ inflammatory monocytes were FACS sorted and pooled per genotype and total RNA was isolated. Transcriptome was analyzed using Affymetrix Clariom S microarray comparing IκB_NS_^-/-^ to IκB_NS_^+/+^ monocytes. Differential expression was determined by applying fold change threshold > 2-fold with FDR < 0.05, resulting in 131 significantly regulated transcripts. **(A)** Ratio-Average plot. Number of up/down regulated transcripts are stated and selected gene symbols (up: red, down: green, blue: otherwise) are shown. **(B)** Gene symbol list of regulated transcripts ranked by log_2_ fold change. **(C)** Gene ontology (GO) enrichment analysis for GO category “Biological process” showing terms with FDR < 0.05 and GO level ≥ 8. Percent of genes in reference to the total number of mapped genes within overrepresented GO terms were calculated, hierarchically sorted and color coded for the group of up/down regulated transcripts. Total number of mapped genes per GO term is stated in brackets. **(D)** Gene Set Enrichment analysis of expression-ranked (IκB_NS_^-/-^ vs. IκB_NS_^+/+^) list of all 22206 transcripts on the microarray for hallmark gene sets with FDR < 0.1. NES: normalized enrichment score. **(E)** qRT-Real-time PCR of *Il-6*, *Nos2* and *Il-1b* normalized to *Rps9* expression. **(F)** Absolute cell counts of CD11b^+^Ly6C^high^ inflammatory monocytes in livers of uninfected and day 3 LM infected IκB_NS_^-/-^ and IκB_NS_^+/+^ mice (n = 6-8). Data are from two independent experiments. Mean ± SEM is shown.

To further characterize the function of IκB_NS_ in the inflammatory response of monocytes to infectious triggers, IκB_NS_^+/+^ and IκB_NS_^-/-^mice were infected with Lm followed by FACS-based isolation of hepatic Ly6C^high^ monocytes on day 3 post infection and subsequent transcriptional profiling. Comparison of Ly6C^high^ monocytes from IκB_NS_^+/+^ and IκB_NS_^-/-^ mice revealed 48 significantly up- and 83 significantly down-regulated transcripts, respectively (**Figure 4A**). As expected, Ly6C^high^ monocytes from IκB_NS_^-/-^ mice showed a striking reduction in the expression of IκB_NS_ transcript (*Nfkbid*) (fold change: -126 compared to IκB_NS_^+/+^), confirming absence of IκB_NS_ in IκB_NS_^-/-^ monocytes. Next to *Nfkbid*, the top 5 down-regulated transcripts were: *Dmkn* (Dermokine, fold change: -24), *Fpr1* (Formyl peptide receptor 1, fold change: -24), *Nos2* (fold change: -21), *Cxcr2* (Chemokine C-X-C motif receptor 2, fold change: -12) and *Ms4a3* (Membrane-spanning 4-domains subfamily A member 3, fold change: -12). The top 5 transcripts with increased expression in Ly6C^high^ monocytes from IκB_NS_^-/-^ mice were: *Id3* (Inhibitor of DNA binding 3, fold change: 13), *Cyp27a1* (Cytochrome P450 family 27 subfamily a polypeptide 1, fold change: 9.3), *Ace* (Angiotensin I converting enzyme 1, fold change: 8), *Hspb1* (Heat shock protein 1, fold change: 7.5) and *Nav1* (Neuron navigator 1, fold change: 5.6). Enrichment analysis of associated GO terms (GO: Biological process) shows involvement of regulated transcripts in inflammatory processes like e.g. mononuclear cell migration, neutrophil migration, regulation of TNF production, regulation of chemokine production and IL-8 production (**Figure 4C**). Interestingly, the vast majority of mapped transcripts from enriched GO terms belong to the group of down-regulated transcripts, thereby implying a dysfunctional inflammatory phenotype of Ly6C^high^ monocytes in IκB_NS_^-/-^ mice. This interpretation is confirmed by Gene Set Enrichment Analysis (GSEA) of hallmark gene sets of the unfiltered microarray dataset containing 22206 transcripts (**Figure 4D**). GSEA of expression ranked transcripts (ranking: IκB_NS_^-/-^ vs. IκB_NS_^+/+^) show significant term enrichment to be associated with diminished expression in IκB_NS_^-/-^ Ly6C^high^ monocytes, consequently resulting in negative normalized enrichment scores (NES). Given the canonical mechanistic function of IκB_NS_ as an inhibitory transcriptional co-factor, the identification of E2F, Myc, and NF-κB transcription factor target gene sets with reduced expression in IκB_NS_^-/-^ Ly6C^high^ monocytes may give further understanding on transcriptional co-targets of IκB_NS_.

Impaired inflammatory phenotype of IκB_NS_^-/-^ *versus* wild type-derived Ly6C^high^ monocytes was further confirmed by RT-PCR analysis comparing expression levels of hallmark transcripts of monocyte-driven inflammation *Il6*, *Nos2* and *Il1b* (**Figure 4E**) which in accordance with the microarray analyses of whole livers (**Figure 1**) were as well downregulated in sorted Ly6C^high^ monocytes from IκB_NS_^-/-^ mice (**Figure 4E**). Of note, absolute numbers of Ly6C^high^ inflammatory monocytes in livers of uninfected and day 3 Lm infected IκB_NS_^+/+^ and IκB_NS_^-/-^mice showed no significant genotype-related differences (**Figure 4F**). Still, both genotypes display solid Lm-induced influx of Ly6C^high^ inflammatory monocytes into the liver by day 3 post Lm infection. In summary, Ly6C^high^ monocytes derived from livers of IκB_NS_^-/-^ mice on day 3 post Lm infection show an impaired inflammatory immune response, yet equal migratory behavior into the liver.

### IκB_NS_-deficiency confers robust protection of mice against lethal *Listeria monocytogenes* infection

**Figure 5:**
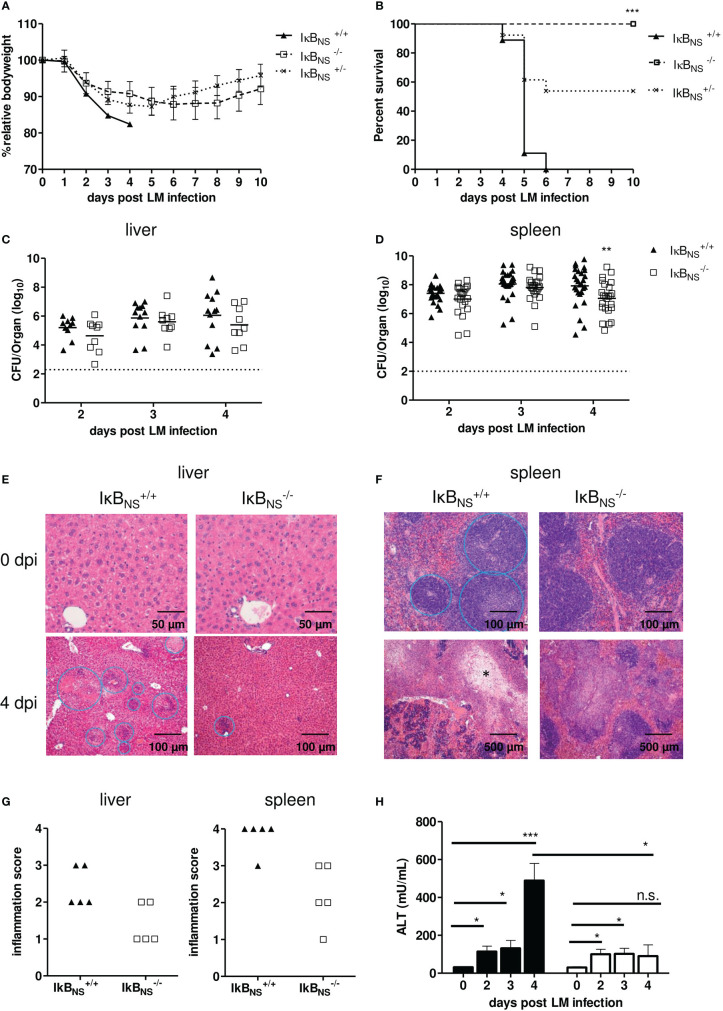
IκB_NS_-deficiency confers robust protection against Lm infection. WT (▲), heterozygous (×) and IκB_NS_-knockout (□) mice were i.v. infected with 10^5^ CFU/mouse *Listeria monocytogenes* and were daily monitored. **(A)** Body weight loss depicted as percent of initial body weight and shown as mean ± SEM. Data are pooled from three independent experiments (IκB_NS_^+/+^: n=9, IκB_NS_^+/-^: n=13, IκB_NS_^-/-^: n=13). **(B)** Kaplan-Meier plot for survival following Lm infection. Statistical analyses were performed using Log-rank (Mantel-Cox) test. *** p=0.0003. **(C)** and **(D)** Colony forming units (CFU) in liver and spleen samples of LM-infected WT and IκB_NS_-knockout mice. Livers of LM-infected IκB_NS_^+/+^ (▲, n= 8-12) and IκB_NS_^-/-^ mice (□, n=9) and spleens from IκB_NS_^+/+^ (▲, n= 25-27) and IκB_NS_^-/-^ mice (□, n=21-24) were sampled at indicated times and homogenized and plated on BHI agar plates. Colonies were counted after 24 h of incubation at 37°C. Data represent log_10_ and the mean per pooled group of three up to five independent experiments is indicated. Dashed lines represent limit of detection for each organ. Statistical analyses were performed using two-way ANOVA with Bonferroni post-test ** p < 0.01. **(E)** and **(F)** Histopathological pictures of liver and spleen samples from uninfected (0 dpi) and Lm infected (4 dpi) mice. After mice were sacrificed, livers and spleens were fixed in 4% PFA. Cut sections were stained with hematoxylin and eosin, and the degree of inflammation was scored in a blinded manner. **(E)**: Upper row: liver sections show normal organ structure. Bottom Row: Leukocytic infiltrations and necrotic foci are encircled. **(F)**: Upper row: spleens show normal architecture with splenic lymphoid sheaths. Bottom row: white pulp is largely replaced by necrosis marked with *. **(G)** Degree of inflammation in liver and spleen samples of IκB_NS_^+/+^ (▲, n= 5) and IκB_NS_^-/-^ mice (□, n=5) scored on day 4 pi. **(H)** Serum level of alanine transaminase (ALT) at different times post Lm infection of IκB_NS_^+/+^ (▲, n=4-5) and IκB_NS_^-/-^ mice (□, n=3-4). Statistical analyses were performed by using two-tailed unpaired student’s t-test (* p< 0.05, ** p< 0.01, *** p< 0.001, n.s., not significant).

As we found IκB_NS_-deficiency to result in a blunted pro-inflammatory response in the entire liver and hepatic Ly6C^high^ monocytes, we next aimed to elucidate IκB_NS_´ overall impact on disease course, pathogen control and organ pathology during Lm infection. To this end, we infected IκB_NS_^+/+^, IκB_NS_^+/-^ and IκB_NS_^-/-^ mice with a high dose (10^5^ CFU) of Lm followed by disease monitoring for up to 10 days. As expected, WT mice reached experimental abortion criteria after 4 to 6 days post infection (**Figures 5A, B**). In stark contrast, 100% of IκB_NS_^-/-^ mice survived the infection, indicating a striking protective effect of IκB_NS_-deficiency against a fatal course of listeriosis. This outcome is causally linked to the IκB_NS_-genotype, since about 50% of heterozygous IκB_NS_^+/-^ mice survived the infection (**Figure 5B**).

Given the fact that IκB_NS_-deficiency resulted in complete protection of mice against an otherwise fatal Lm infection, we next evaluated pathogen growth in infected organs. Unexpectedly, the bacterial burden in liver and spleen on day 2 and 3 post infection was almost identical in WT and IκB_NS_^-/-^ mice (**Figures 5C, D**). On day 4 post infection, however, we observed a significantly improved pathogen clearance in spleens of IκB_NS_^-/-^ mice in comparison to their WT counterparts. For IκB_NS_^-/-^ mice we checked if they cleared the pathogen later on post infection. Indeed, no bacteria were detectable on day 10 post infection, neither in liver nor spleen samples (data not shown), confirming that IκB_NS_^-/-^ mice eventually cleared the pathogen.

**Figure 6:**
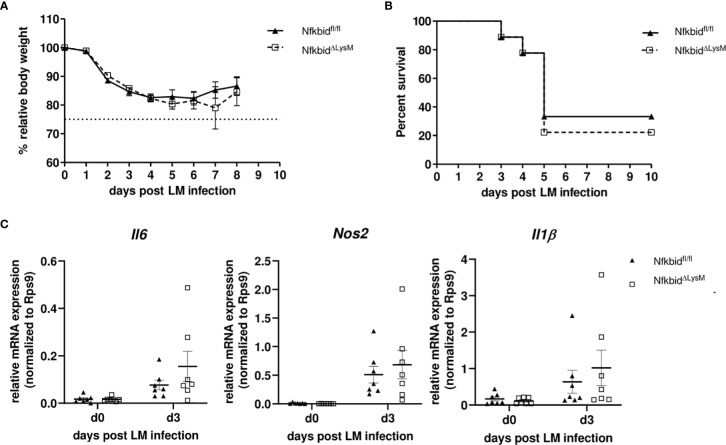
Infection of conditional knock-out mice harboring a specific deletion of IκB_NS_ in macrophages and granulocytes with *Listeria monocytogenes*. **(A)** Body weight loss depicted as percent of initial body weight and shown as mean ± SEM. Data are pooled from three independent experiments (Nfkbid^fl/fl^ littermates (▲, n=9), NfkbidΔ^LysM^ (□, n=9). **(B)** Kaplan-Meier plot for survival following LM infection. **(C)** Relative mRNA expression levels of pro-inflammatory cytokines (*Il6, Nos2, Il1b*) from sorted inflammatory monocytes from liver samples of NfkbidΔ^LysM^ mice and Nfkbid^fl/fl^ littermate controls. Represented is the mean ± SEM from 7 mice per group and point in time from two independent performed experiments.

Next to bacterial burden, we scored severity of inflammation in livers and spleens to evaluate pathogen-induced pathology. As expected, multiple foci with leukocyte aggregates and severe, acute, necrotizing, inflammation-related structural changes were observed in livers of susceptible WT mice (**Figure 5E**, left). Compared to that, the number of infectious foci was clearly reduced in livers of IκB_NS_^-/-^ mice (**Figure 5E**, right). In spleens, we as well observed severe immunopathology in WT mice. Here, the splenic structure of the white pulp was almost completely destroyed and multiple necrotic areas became visible. Again, a significantly reduced immunopathology was seen in spleens of IκB_NS_^-/-^ mice (**Figure 5F**, right). Scoring of pathology and inflammatory degree confirmed the notion of an ameliorated inflammatory state in livers and spleens from IκB_NS_^-/-^ mice compared to their WT counterparts (**Figure 5G**). Another indicator of liver pathology is the serum concentration of the liver enzyme alanine amino-transferase (ALT), passively sequestered by necrotic hepatocytes. As expected, and confirming the histological results, the ALT level increases until day 4 post infection in WT mice, while it remained constantly low in IκB_NS_^-/-^ mice (**Figure 5H**). Thus, the absence of an exaggerated innate immune response in IκB_NS_^-/-^ mice obviously protects mice from organ damage, ultimately conferring robust protection against lethal Lm infection. To further prove, whether protection could be attributed directly to an altered transcriptional program in monocytes, we infected Nfkbid^ΔLysM^ mice lacking IκB_NS_ specifically in myeloid Ly6M-expressing cells including Ly6C^high^ monocytes. As shown in **Figures 6A, B**, lack of IκB_NS_ exclusively in myeloid cells did not confer protection against Lm infection, indicative for suggesting a myeloid cell-extrinsic rather than -intrinsic effect of IκB_NS_. This was further supported by quantification of the expression of selected hallmark genes of monocyte-driven inflammation in sorted Ly6C^high^ monocytes from livers of Nfkbid^ΔLysM^ mice and respective littermate controls that did not reveal any differences between the genotypes (**Figure 6C**).

### IκB_NS_^-/-^ mice are impaired in IL-10 production both in the steady state and during *Listeria monocytogenes* infection

**Figure 7:**
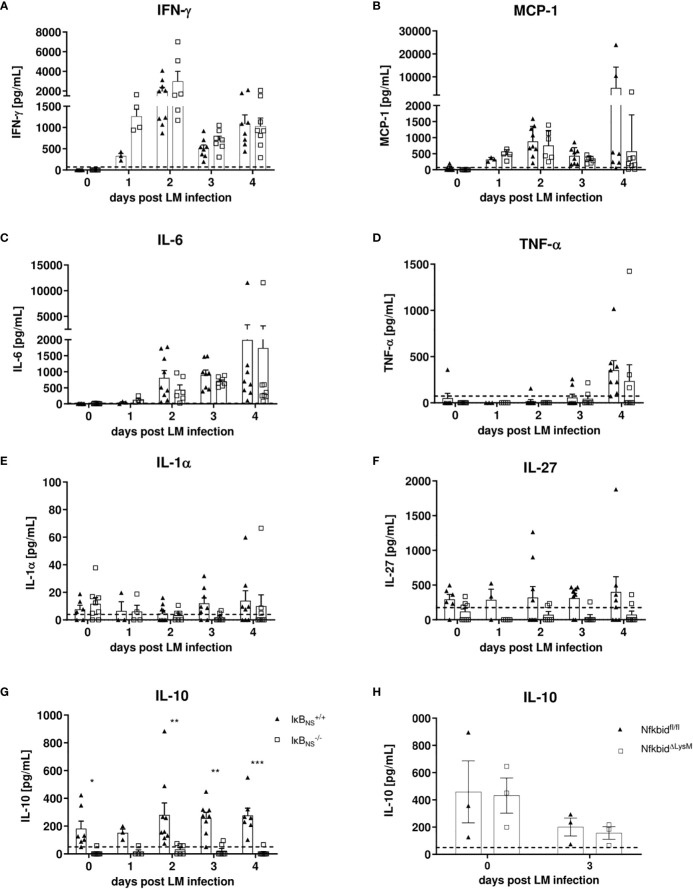
Analysis of pro-inflammatory mediators in serum samples during the course of Lm infection. IκB_NS_^+/+^ and IκB_NS_^-/-^ mice were infected with 10^5^ CFU Lm and heart blood was collected at the indicated times and sera was collected and used for detection of pro-inflammatory mediators *via* cytometric bead array. Shown are the mean ± SEM from **(A)** IFN-γ, **(B)** MCP-1, **(C)** IL-6, **(D)** TNF-α, **(E)** IL-1α, **(F)** IL-27 and **(G)** IL-10 from n=7-9 mice pooled from two independent performed experiments. **(H)** Serum IL-10 analysis of Lm infected NfkbidΔ^LysM^ mice and Nfkbid^fl/fl^ littermate controls mice. Statistical analyses were performed with two-way ANOVA with Bonferroni post-hoc test. *p < 0.05, **p < 0.01, ***p < 0.001.

Since we largely excluded a myeloid cell-intrinsic mechanism being responsible for the blunted innate immune response in IκB_NS_^-/-^ mice, we next took a closer look on the inflammatory milieu being induced following Lm infection that might either directly or indirectly affect monocyte activation and differentiation during *in vivo* infections. For this purpose, we performed proteome profiling of serum cytokines related to anti-bacterial responses during the course of infection. Of the analyzed cytokines, IFN-γ, MCP-1, IL-6, TNF-α, IL-1α, IL-27 and IL-10 were detectable at reliable concentrations in sera of Lm infected mice (**Figures 7A–G**). Interestingly, despite a (statistically not significant) trend for higher basal level expression of IL-27 in WT mice, no significant genotype-specific differences were observed for the majority of cytokines. Strikingly though, IκB_NS_^-/-^ virtually lack serum IL-10 in the steady state and fail to produce it during Lm infection (**Figure 7G**). Of note, well in line with the normal course of infection in conditional Nfkbid^ΔLysM^ mice (**Figures 6A, B**), IL-10 levels (**Figure 7H**) as well as any other cytokine tested (data not shown) were not affected by IκB_NS_-deficiency exclusively in myeloid cells. We also quantified *Il10* gene expression in livers of WT and IκB_NS_^-/-^ mice, confirming lack of IL-10 expression in the absence of IκB_NS_ (data not shown). Since we demonstrated previously that *Il10* is a direct target gene of IκBNS in Th17 cells (8), it is most likely that this is the case as well in other IL-10-producing cell subsets. In any case, the striking IκB_NS_-dependent reduced IL-10 level most likely contributes to an altered cytokine milieu potentially affecting the priming and differentiation of Ly6C^high^ monocytes in the Lm infected host.

## Discussion

Currently, five atypical IκB’s have been described: Bcl-3, IκB_ζ_, IκB_NS_, IκB_η_ and IκB_L_ (reviewed in (3, 26) Among these, IκB_NS_ and IκBη are structurally most related to one another, as both have similar numbers of ankyrin repeat domains and do not feature a Transcription Activation Domain (TAD). All IκBs are generally thought to influence NF-κB-dependent transcriptional regulation in the nucleus and many inflammatory conditions encompass NF-κB-signaling within various immune and non-immune cell types. Consequently, one might expect that deficiency in atypical IκBs would affect the outcome of sterile and non-sterile inflammation. However, there is no common sense to date whether atypical IκB would generally boost or ameliorate inflammation. Moreover, while there is still little systematic knowledge regarding the level of IκBs´ expression in different immune cell types and their according roles in inflammatory conditions with different etiologies, there is evidence for IκB-type-specific influences on common inflammatory NF-κB-target genes (27). Data obtained in our study identified IκB_NS_ as potential driver of inflammation in murine listeriosis, since mice deficient for IκB_NS_ are protected from the development of fatal hyper-inflammation and lethal immune pathology. This finding was somehow unexpected in light of a previously published study showing that IκB_NS_ is involved in the selective inhibition of a couple of MyD88-dependent genes (e.g. as *Il6*, *Il12p40* and *Il18*) following LPS-stimulation, thereby preventing excessive inflammation (14). While these data were obtained in macrophages in an *in vitro* setting and therefore did not take into account the micro milieu in which macrophage activation would occur, the authors also demonstrated a high susceptibility of IκB_NS_^-/-^ mice for the development of endotoxin shock following intraperitoneal (i.p.) LPS injection.

In contrast to i.p. LPS injection that would primarily target peritoneal macrophages, we used systemic Lm infection. Following intravenous injection, the pathogen spreads *via* the circulation throughout the whole organism with liver and spleen representing major replication sites. In the liver, IκB_NS_-deficiency was associated with pronounced transcriptional alterations on day 3 and 4 post infection, but in stark contrast to the LPS challenge, lack of IκB_NS_ was associated with a hypoinflammatory gene expression profile and marked resistance to Lm infection. More specifically, especially transcripts associated with an inflammatory myeloid cell phenotype and function were found to depend on IκB_NS_. It is well-known that Ly6C^high^ monocytes play an important role during Lm infection starting with their rapid and CCL2-mediated recruitment and CCR2-dependet migration into the liver, where they produce amongst others TNF and iNOS for antimicrobial defense (28). Recruited monocytes are able to differentiate into TNF- and iNOS-producing DCs (Tip-DCs) that have been described to contribute to bacterial clearance (29). Mice lacking CCL-2 or its receptor CCR-2 are characterized by a reduced Tip-DC frequency in Lm-infected spleens resulting in uncontrolled bacterial growth (30), thus underlining the importance of monocyte-derived dendritic cells, originating from Ly6C^high^ monocytes, for the outcome of infection. Interestingly, we found reduced *Ccl2* expression in IκB_NS_^-/-^ livers and also a reduced expression of *Ccl1* in Ly6C^high^ monocytes derived from Lm infected IκB_NS_^-/-^ mice, while the frequency of Ly6C^high^ monocytes within the liver was not affected (**Figure 4F**). Moreover, the expression level of hallmark pro-inflammatory mediators such as *Il6*, *Nos2* and *Il1b* was markedly reduced in IκB_NS_-deficient mice as observed in whole liver tissue (**Figure 1C**) as well as in sorted liver Ly6C^high^ monocytes (**Figure 4E**) compared to WT counterparts. Surprisingly, and in contrast to previous data by Kuwata et al. showing that IκB_NS_ limits IL6 production in LPS-stimulated macrophages (14), we found higher *Il6* levels in wild type compared to IκB_NS_ knock out mice, suggesting that during listeriosis the inflammatory environment and the interplay of different immune cell subsets might influence the regulation of NF-κB-dependent genes by IκB_NS_. Interestingly, on day 3 post infection (**Figure 2B**, centre) also a group of transcripts emerges, that mainly annotates to the huge family of olfactory receptors (Olfr) and shows overall reduced expression in IκB_NS_^-/-^ compared to wild type livers. Functional involvement of olfactory receptors in non-sensory tissues only recently became evident and in the liver it is thought to have chemosensory purposes regulating homeostasis (31) and mediating glucose metabolism (32). Another aspect that should be considered is the impact of non-hematopoietic cells during Lm infection and here especially hepatocytes. In our study we were mainly focused on hematopoietic cells since we detected strong *Nfkbid* promotor activity in myeloid cells suggesting an important role of IκB_NS_ in those subsets. Effector mechanism in the liver early after infection are orchestrated by a complex network of liver resident cells including hepatocytes and Kupffer cells and the infiltrating subsets such as Ly6C^high^ monocytes, NK cells and neutrophils (33). Moreover, it is known that Ly6C^high^ monocytes are subjected to a high plasticity with the capability to switch their phenotype towards a restorative Ly6C^low^ phenotype. This switch can be mediated by phagocytosis and exposure to certain cytokines such as IL-4 or IL-33 derived from necrotic Kupffer cells (34). Future studies will help to understand the plasticity of Ly6C monocytes in this context. However, another interesting aspect is the remaining high expression of *Nfkbid* in Ly6C^high^ monocytes during the course of infection. The IκB_NS_ protein was shown to be rapidly degraded (35) upon induction. In our study we found the transcript constantly high expressed for several days after infection. Several studies showed IκB_NS_ to be induced upon stimulation (8, 13, 14) and it is conceivable, that a systemic inflammation leads to systemic induction of IκB_NS_ in a variety of immune cells, ultimately resulting in an imbalanced turn-over. In this context, it would be of interest to analyze the kinetics of IκB_NS_ expression on the protein level. Indeed, we performed Western Blot analysis of IκB_NS_ protein expression in the liver at different times post infection (data not shown), but probably due to the high abundance of digestive enzymes and proteases in liver tissue, we did not detect meaningful contents of IκB_NS_ protein. Of note, there is still very limited general information regarding the nuclear IκB_NS_ protein. For T cells it was shown that upon its induction IκB_NS_ is detectable as a 70 kDa protein in the nucleus, while a 35 kDa form resides in the cytoplasm (6). While we can only speculate about the protein turnover in Ly6C ^high^ monocytes, our unbiased transcriptomic approach identified a potential role of IκB_NS_ especially in myeloid cell activation and function during listeriosis. Indeed, we could confirm that Ly6C^high^ monocytes are dependent on IκB_NS_ as evidenced by a blunted and generally delayed pro-inflammatory signature in the absence of IκB_NS_ (**Figure 4**). This delay but not complete absence of innate immune activation might explain why IκB_NS_^-/-^ mice are still able to clear the pathogen (**Figures 5C, D**).

We showed that the blunted response of Ly6C^high^ monocytes to infection was not evident in Nfkbid^ΔLysM^ mice (**Figure 6**). In this context we discovered that IκB_NS_-deficient mice fail to produce any significant quantities of IL-10, both in the steady state and during infection (**Figure 7**). At a first glance this is counterintuitive since IL-10 is generally considered immunosuppressive and a potent anti-inflammatory inhibitor capable to suppress the production of other pro-inflammatory cytokines. However, there is evidence that IL-10, under certain conditions, can have pro-inflammatory capacities as well (36). Moreover, *Il10*-deficient mice are highly resistant to Lm infection and this resistance is associated with an increased pro-inflammatory response by macrophages and NK cells, improved pathogen clearance as well as reduced immunopathology in liver tissue compared to control mice (37). The fact that we observed stable IL-10 levels in WT mice already in the steady state suggests that conditioning by endogenous IL-10 might affect the priming of effector cells during infection. It is known that IL-10 influences the differentiation of circulating blood monocytes into different types of tissue macrophages (38). In the inflamed peritoneum, IL-10 constitutes a dominant mediator directing infiltrating monocytes into a certain macrophage phenotype. In the early phase of infection, neutrophils were shown to be a dominant source of IL-10 and that increased levels of IL-10 might provoke the formation of different macrophage phenotypes. Moreover, IL-10 was suggested to be a key cytokine regulating the developmental fate of monocytes in the infected peritoneum (39). It is tempting to speculate that this phenomenon might also be true for monocyte differentiation in the inflamed liver tissue during murine listeriosis. Indeed, it has been already described that IκB_NS_ plays important roles in the development of IL-10-competent B cells and that it is involved in the production of IL-10 by B cells (40). Moreover, recent reports described that marginal zone B cell-derived IL-10 increases the susceptibility to Lm infection (41). Importantly, we have shown previously that stimulated Th17 cells show an increased binding of IκB_NS_ to the *Il10* gene locus compared to unstimulated Th17 cells and IκB_NS_-deficient controls suggesting that IL-10 is a direct target gene of IκB_NS_ (8). The direct NF-κB interaction partner that mediates increased binding to the *Il10* gene promoter remains elusive, because it is known that IκB_NS_ itself has no own DNA binding domain. With regard to Lm infection, Th17 cells are not the main population involved in combating the infection, but it might be conceivable that other IL-10 producing cells are directly affected by IκB_NS._ To experimentally address whether serum IL-10 would affect the outcome of infection, we administered recombinant IL-10 into Lm-infected IκB_NS_-deficient mice (200 ng IL-10 i.v. one day prior Lm infection and on each consecutive day until day 5 post infection). However, IL-10 complementation at this time did not affect disease severity (data not shown). These data suggest, that either long-term conditioning of immune cells by physiological IL-10 levels or yet unknown and potentially multifaceted mechanisms contribute to the striking protection from fatal courses of listeriosis in mice lacking IκB_NS_.

In conclusion we describe here a crucial role for IκB_NS_ during Lm infection. IκB_NS_-deficient mice showed a blunted pro-inflammatory immune response and especially a reduced pro-inflammatory signature in Ly6C^high^ monocytes. This resulted in reduced immunopathology in spleen and liver and moreover, in protection of mice against lethal outcome of Lm infection. The observed effects are not intrinsically mediated by IκB_NS_ in monocytes, but it seems that IκB_NS_-deficiency alters the immunological environment in such a way that the priming of effector mediators is modified. Furthermore, we found that IκB_NS_-deficient mice virtually lack IL-10 which might result in dysregulated priming of effector cells as a potential mechanism underlying protection against an otherwise fatal Lm infection. Future studies should focus on the molecular mechanism underlying the blunted innate immune response in the absence of IκB_NS_. In any case, as we identified IκB_NS_ as driver of inflammation and immunopathology in infection-associated inflammation, it may constitute an interesting pharmaceutical target to prevent exaggerated pro-inflammatory immunity and severe outcome of infectious diseases.

## Data availability statement

Microarray data presented in the study are deposited in National Center for Biotechnology Information’s (NCBI’s) Gene Expression Omnibus (GEO) and are accessible through the GEO series accession numbers GSE220267.

## Ethics statement

The animal study was reviewed and approved by Landesverwaltungsamt Sachsen-Anhalt - Verbraucherschutz, Veterinärangelegenheiten.

## Author contributions

SF, AJ, JV and AP carried out the experiments. OK performed the histopathological analysis. SF, AJ and DB wrote the manuscript. SF, AJ, IS and DB conceived the study, planned the experiments and revised the manuscript. All authors contributed to the article and approved the submitted version.
